# Supplementary material for: Biosensing with the singular phase of an ultrathin metal-dielectric nanophotonic cavity
Source: Nat Commun. 2018 Jan 25;9:369. doi: 10.1038/s41467-018-02860-6 (PMC5785542; doi:10.1038/s41467-018-02860-6)
Supplement: Supplementary file 1 — Supplementary Information [file 41467_2018_2860_MOESM1_ESM.pdf]

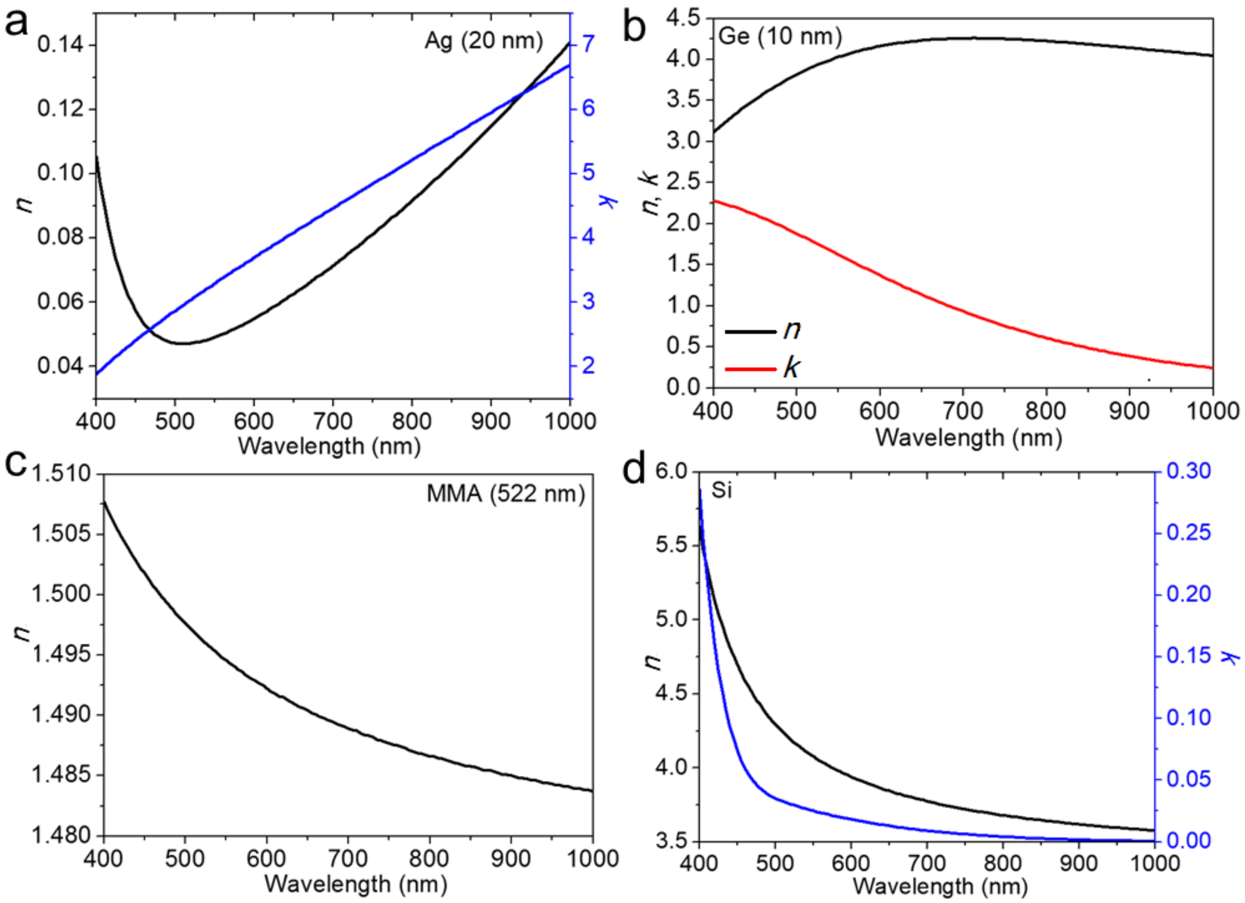

Supplementary Figure 1 | Measured optical constants (*n* & *k*) of thin films. (a) Ag, (b) Ge, (c) MMA, and (d) Si.

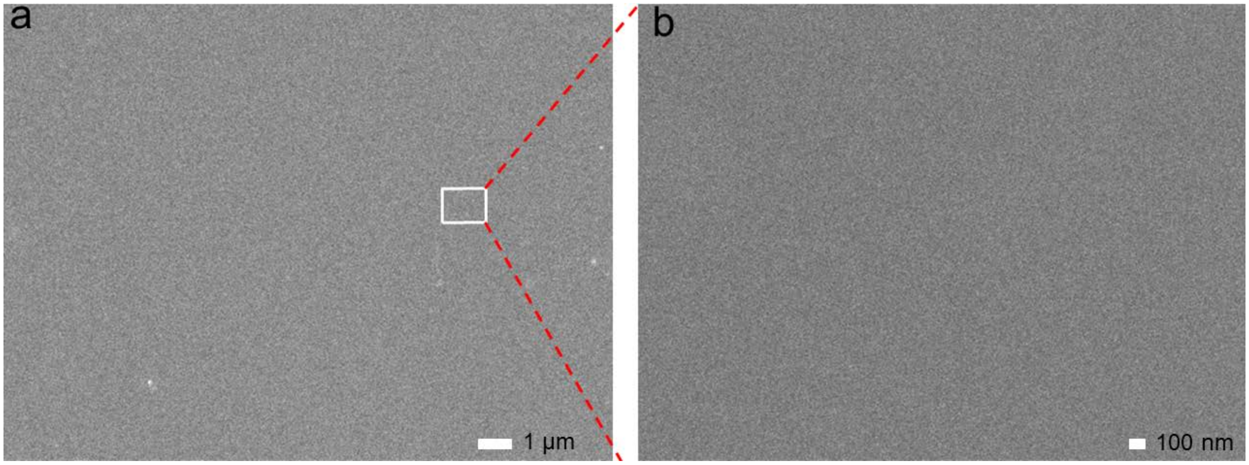

Supplementary Figure 2 | FE-SEM images of thermally evaporated 10 nm thick Ge film. (a) panoramic view and (b) high resolution image**.**

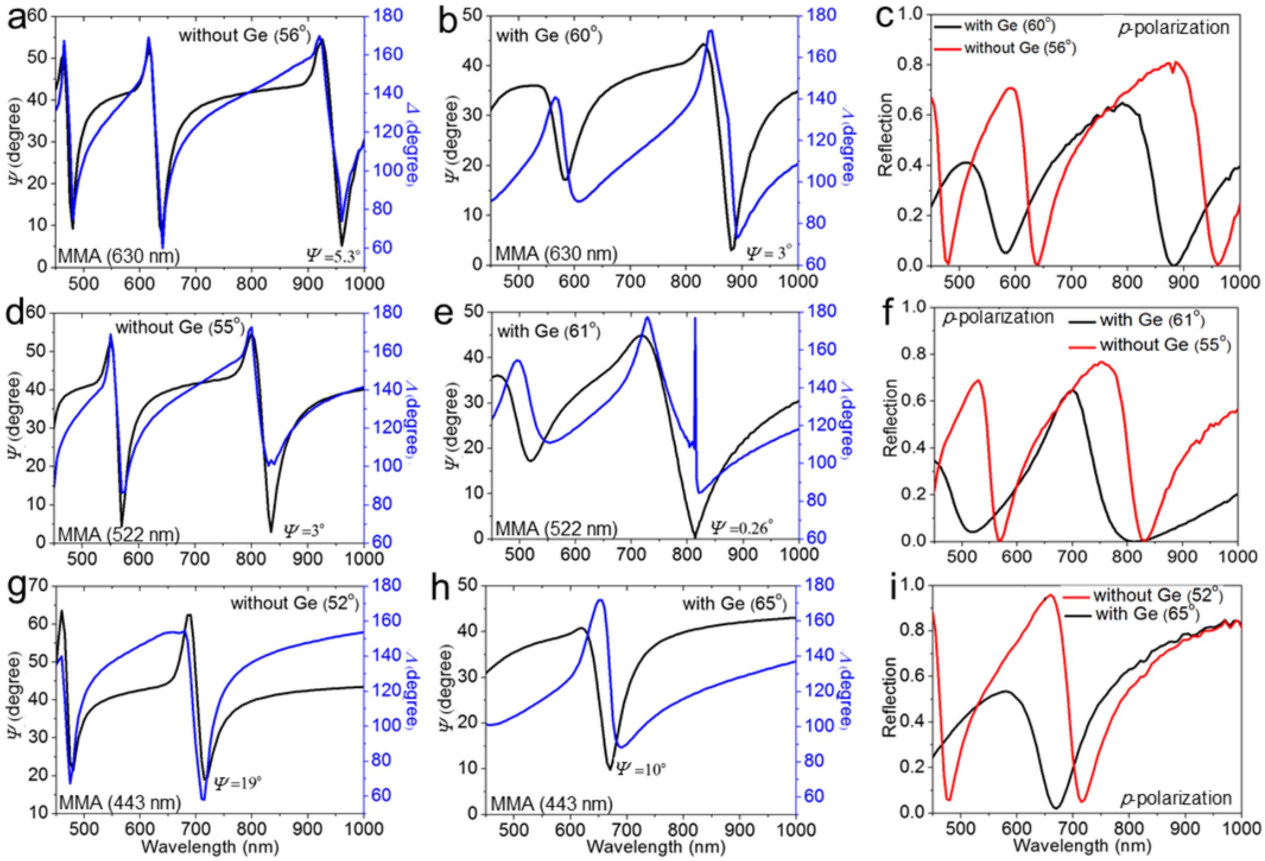

Supplementary Figure 3 | Experimentally determined ellipsometry parameters (ψ and Δ) and *p*-polarized reflection spectra of samples with and without Ge layer. (a) to (c) for 630 nm MMA layer, (d) to (f) for 522 nm MMA layer and (g) to (i) for 443 nm MMA layer. The incident angle is fixed where ψ_{min} is obtained for longer wavelength mode.

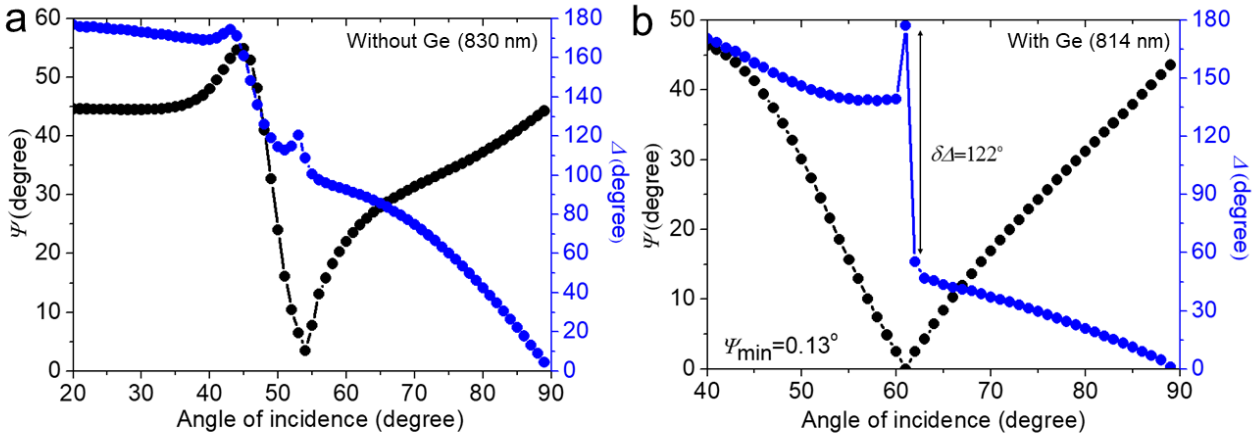

Supplementary Figure 4 | Measured pair of ellipsometry parameters as a function of incident angle. For (a) without Ge sample at 835 nm and (b) with Ge sample at 814 nm.

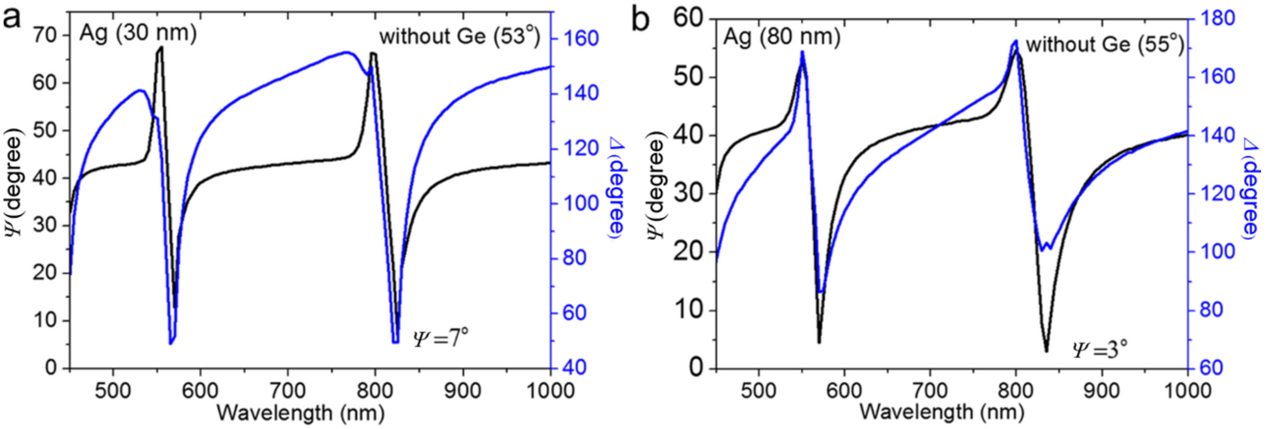

Supplementary Figure 5 | Measured ψ and Δ spectra of without Ge sample at lowest ψ angle for different thickness of bottom Ag layer. (a) for 30 nm and (b) for 80 nm.

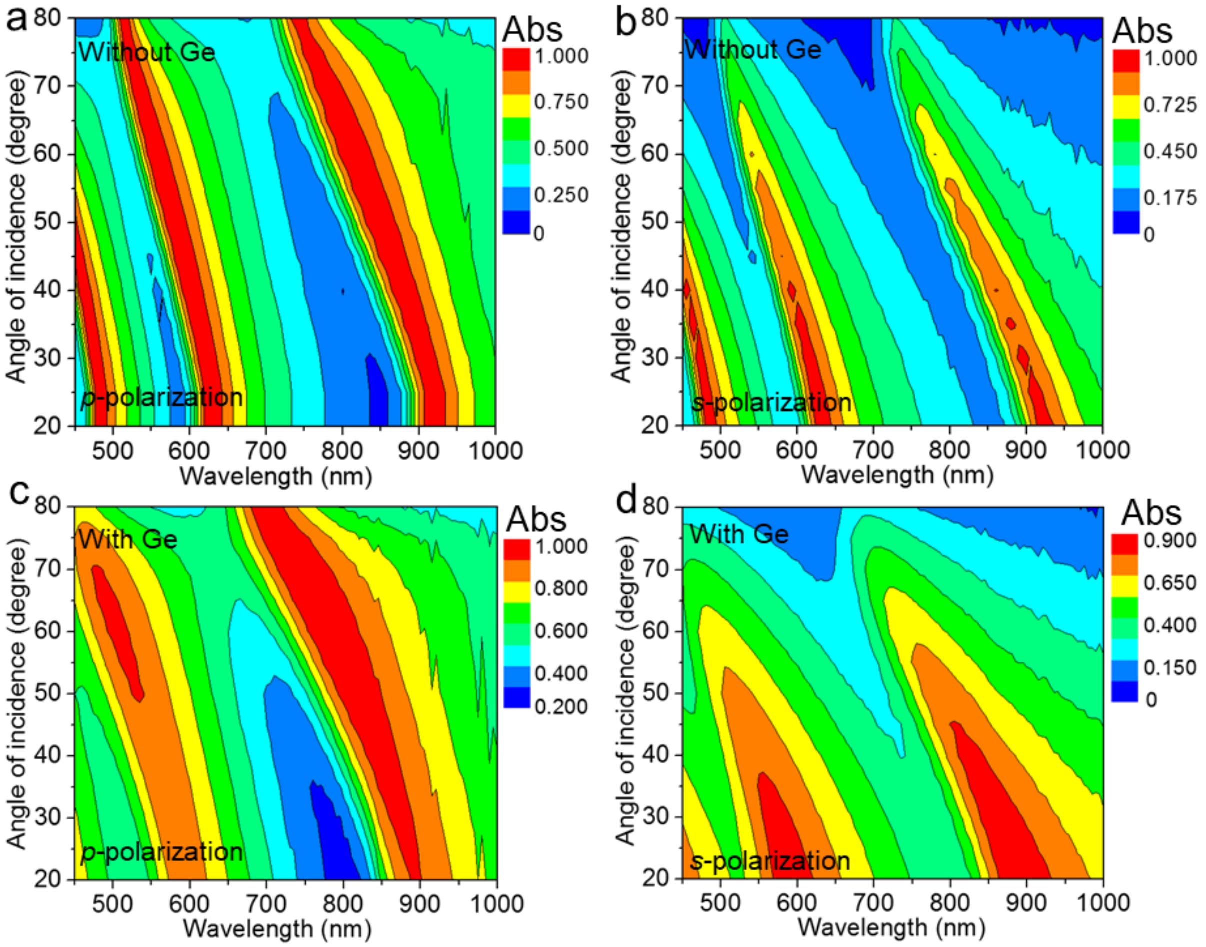

Supplementary Figure 6 | Experimental angular and polarization depended absorption spectra of without Ge and with Ge samples. Absorption spectra of without Ge sample (a) *p*-polarization, and (b) *s*-polarization. Absorption spectra of with Ge sample (c) *p*-polarization, and (d) *s*-polarization.

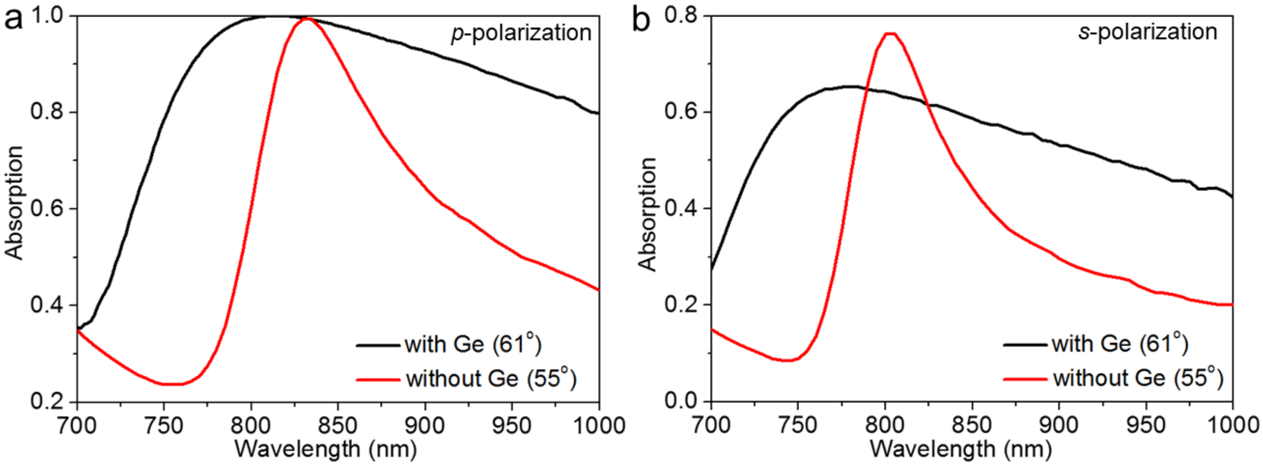

Supplementary Figure 7 | Absorption spectra of with Ge and without Ge samples at $\psi_{\min}$ angle. (a) for *p*-polarization and (b) for *s*-polarization.

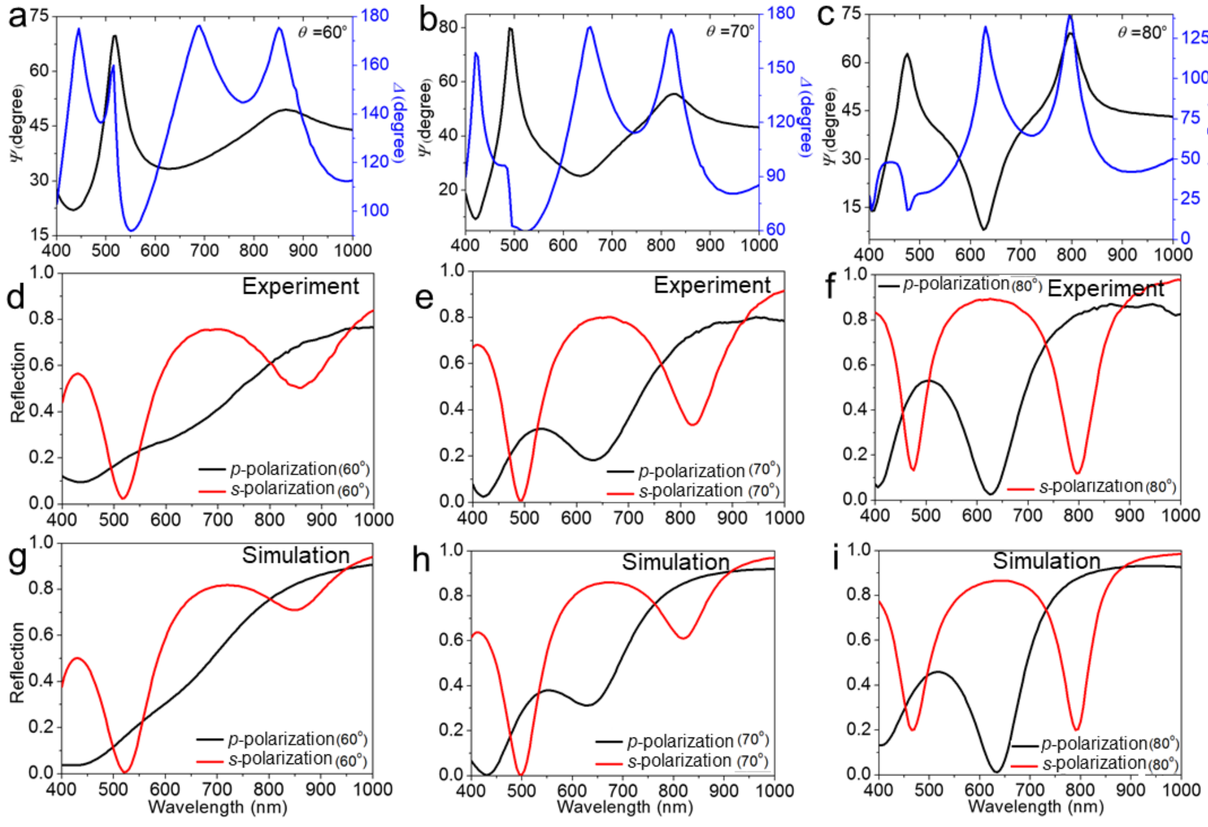

Supplementary Figure 8 | Ellipsometry parameters (*ψ* and *Δ*) and *p*- and *s*-polarized reflection spectra of proposed sample without top Ag layer for different angles of incidence. (a)-(c) Ellipsometry parameters. (d)-(f) Experimental reflection spectra for *p*- and *s*-polarization and (g)-(i) Simulated reflection spectra for *p*- and *s*-polarization.

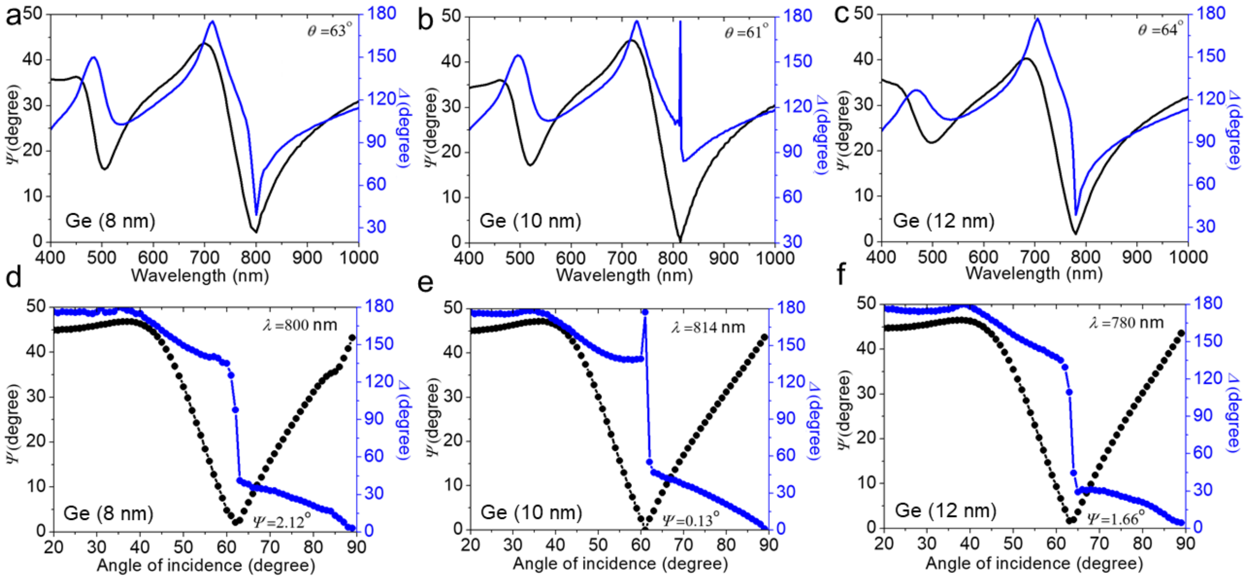

Supplementary Figure 9 | Measured ψ and Δ spectra for different thickness of Ge layer. For wavelength scan (a) 8 nm, (b) 10 nm, (c) 12 nm and for angular scan (d) 8 nm, (e) 10 nm, (f) 12 nm. The corresponding incident angle and excitation wavelength is shown in each figure.

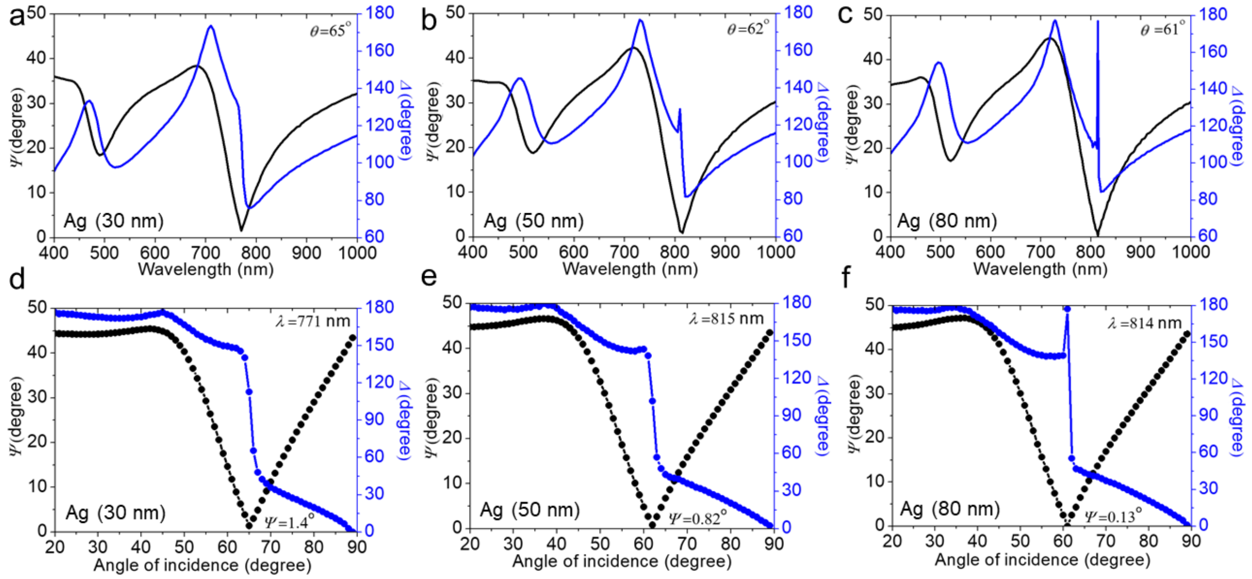

Supplementary Figure 10 | Measured ψ and Δ spectra for different thickness of bottom Ag layer. For wavelength scan (a) 30 nm, (b) 50 nm, (c) 80 nm and for angular scan (d) 30 nm, (e) 50 nm, (f) 80 nm. The corresponding incident angle and excitation wavelength is shown in each figure.

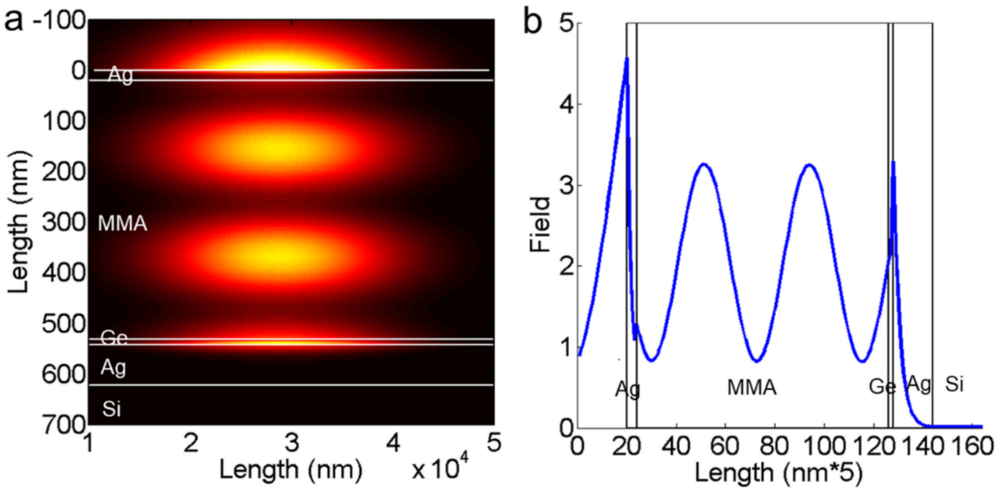

Supplementary Figure 11 | Field map (a) Calculated field distribution along the four-layered system for the mode at 520 nm wavelength and 61° angle of incidence, and (b) its magnitude as a function of depth along the multilayer.

Supplementary Note 1: Optical constants of thin films

Variable angle high-resolution spectroscopic ellipsometry (J. A. Woollam Co., Inc, V-VASE) was used to obtain the optical constants and thickness of Ag, Ge, and MMA. By means of thermal evaporation of Ag pellets, Ag thickness of 20 nm and 80 nm was deposited on a Si substrate and an oscillator model was used to fit the measured ellipsometry data. For the measurements of Ge thin film, 10 nm thick Ge layer was deposited on a Si substrate using thermal evaporation of Ge pellets. An oscillator model was used to fit the measured data to obtain the complex refractive index of Ge. In the case of MMA, MMA copolymer was spin coated at 3000 r.p.m. on a Si substrate and Cauchy model was used to obtain n & k values of MMA. However, complex refractive indices of substrate (Si) were directly taken from the V-VASE software. The obtained optical constants of Ag, Ge, MMA and Si are shown in supplementary Fig. 1.

Supplementary Note 2: Spectroscopic ellipsometry characterizations of the fabricated samples

In this section, we present a detailed explanation for the interesting phase singularity behavior observed for the proposed sample by comparing the results of various samples (with and without Ge layer).

Initially, we have optimized the thickness of cavity (MMA) layer to achieve minimum ψ and maximum phase change. For this purpose, we have first determined the thickness of MMA layer by spin coating MMA on Si wafer at different r. p. m. Ellipsometrically determined thickness of MMA layer at 2000, 3000 and 4000 r. p. m. are ~630 nm, ~522 nm and ~443 nm, respectively. Thickness of other layers in the multilayer are: top Ag layer (20 nm), Ge layer (10 nm) and bottom Ag layer (80 nm). Supplementary Fig. 3 represents the experimental

ellipsometry spectra (ψ and Δ) and *p*-polarized reflection spectra of samples with and without Ge layer for different MMA layer thickness. All spectra were plotted for an incident angle at which minimum ψ was obtained for longer wavelength mode.

Importantly, a singular phase behavior at ψ_{min} point and blue shift in resonance was obtained for all thickness of MMA layer by introducing a thin Ge layer (10 nm) in the geometry. It shows that phase singularity is only possible when Ge layer is present in the geometry. One can see that the modes were blue shifted by decreasing the thickness of MMA layer and lowest ψ value, and minimum *p*-polarized reflection were obtained for MMA layer of thickness 522 nm (Figs. d to f). For 522 nm thick MMA layer, obtained ψ_{min} for longer wavelength mode (above 800 nm) is 3° (R=0.008 at 835 nm) for without Ge sample and 0.26° (R= 0.0000114 at 814 nm) for with Ge sample. In contrast to without Ge sample, ψ_{min} is reduced 11 times (reflection reduced to 725 times) and a sharp change in phase is obtained by using with Ge sample. It should be noted that reduction in ψ_{min} and higher phase change at ψ_{min} occurs for angular scan as compared to wavelength scan. Therefore, in supplementary Fig. 4, we plot the ellipsometry parameters of samples without and with Ge layer, as a function of incident angle. One can see that ψ_{min} was exactly obtained at the minimum incident angle for both samples (55° for without Ge and 61° for with Ge). It is evident from the results that with Ge sample only shows an abrupt phase change at ψ_{min} point (122° phase change). The obtained ψ_{min} using angular scan was 0.13° (23 times reduction compared to without Ge sample) and the calculated FOM (1/ ψ_{min}, where ψ is expressed in radians) is 445, which is the highest value reported so far based on singular phase concept. It indicates that a thin layer of Ge plays an important role in the geometry to achieve point of darkness and phase singularity.

We have further studied the ellipsometry parameters by changing the thickness of bottom Ag layer for without Ge sample. As shown in supplementary Fig. 5, no considerable singular phase behavior was observed by changing the thickness of bottom Ag layer thickness from 20 nm to 100 nm. Here, MMA layer and top Ag layer thickness were set to 552 nm and 20 nm, respectively. We confirmed that no phase singularity is possible for without Ge sample by optimizing the combination of silver and dielectric (MMA) thickness. Therefore, the physical mechanism of achieving singular phase in the proposed system is only due to the presence of Ge thin film.

We then experimentally investigated the influence of incident angle and polarization states on absorption (Abs=1-R, where T=0) spectra of without Ge and with Ge samples. The absorption spectra as a function of incident angle (20° to 80°) and polarization states (*p*- and *s*-polarization) are shown in supplementary Fig. 6. In the case of without Ge sample, for *p*-polarization (supplementary Fig. 6a), almost perfect absorption was obtained for all angles of incidence and narrow band perfect absorption was obtained for *s*-polarization (supplementary Fig. 6b) at lower incident angles (below 35°). For with Ge sample (supplementary Fig. 6c), a wide band perfect absorption for longer wavelength mode at higher incident angle is obtained for *p*-polarization. However, no perfect absorption was observed for *s*-polarization (supplementary Fig. 6d) even at lower incident angles. In particular, *p*-polarized absorption increased (supplementary Fig. 7a) and *s*-polarized absorption decreased (supplementary Fig. 7b) for with Ge sample as compared to without Ge sample. The *p*- and *s*-polarized absorption spectra recorded at ψ_{min} angle of with Ge and without Ge samples are shown in supplementary Fig. 7. Since ψ_{min} is obtained at 814 nm for with Ge sample, the measured *p*- and *s*-polarized absorption at 814 nm wavelength are 99.999% and 63%, respectively. On the other hand, *p*-polarized

reflection decreased and *s*-polarized reflection increased after introducing a thin Ge layer in the multilayer.

This is the reason for with Ge samples to show reduced $\psi_{\min}$ and sharp singularity as compared to without Ge samples because $\tan\Psi = \left|\frac{r_p}{r_s}\right|$. In supplementary Fig. 8, we show the results of proposed sample without top Ag layer. This sample shows totally different spectra compared to other three samples (Fig. 1a to 1c in the manuscript). Interestingly, a reflection peak instead of reflection dip was obtained in ψ spectrum. In order to clearly understand this, we have recorded the ellipsometry parameters (ψ and Δ) and *p*- and *s*-polarized reflection spectrum at higher angles of incidence (60° to 80°). In supplementary Fig. 8, we present the results of this sample for different angles of incidence. As shown in supplementary Fig. 8 (a-c), the cavity modes were converted into reflection peaks in ψ spectra. This is because the *s*-polarized light provides minimum reflection at that spectral wavelength as compared *p*-polarized light, which is shown in supplementary Fig. 8 (d-f). However, for other three samples (Fig. 1a, b & c in the manuscript), *p*-polarized light provides the minimum reflection at the resonance wavelengths as compared to *s*-polarized light, so that reflection dip was obtained. A blue shift in resonance wavelength was obtained with increase in angle of incidence, which is similar to the case with other three samples. Also, at a particular incident angle (70°) higher quality factor mode with maximum intensity was obtained at shorter wavelength, which is around 495 nm. Interestingly, *s*-polarized reflection is almost zero at that wavelength (supplementary Fig. 8e). That is why a sharp reflection peak was obtained at 495 nm wavelength. In short, the proposed sample (Fig. 1c in the manuscript) provides zero reflection for *p*-polarized light at a particular angle of incidence for longer wavelength mode so that lowest ψ is obtained for that mode. However, the sample

without top Ag layer (Fig. 1c in the manuscript) provides zero reflection for *s*-polarized light at a particular angle of incidence for the shorter wavelength mode so that highest ψ is obtained for that mode. This behavior is because $\tan\Psi = \left|\frac{r_p}{r_s}\right|$. The physical mechanism of achieving almost zero reflection for *s*-polarized light using the proposed sample without top Ag layer could be due to generalized Brewster angle (pseudo) effect[1]. We further calculated *p*- and *s*-polarized reflection spectra of this sample for different angles of incidence using TMM (supplementary Fig. 8 (g-i)), which were in very good agreement with experimental reflection spectra.

We have further investigated the Ge layer thickness on achieving phase singularity at ψ_{min}. In supplementary Fig. 9, we present the wavelength and angular scan results of ellipsometry parameters by slightly varying the thickness (8 nm-12 nm) of Ge layer. As can be seen, lowest ψ_{min} and maximum phase change was obtained for 10 nm Ge thickness. We noticed that phase change is only significant when Ge thickness is within 8-12 nm. We have also investigated the phase singularity variation by changing the thickness of top and bottom Ag film thickness. It should be noted that top Ag film thickness should be within 18-21 nm range to obtain significant phase singularity. However, phase singularity was observed for different thickness of bottom Ag film (20 nm to 100 nm) and maximum phase change was obtained for 80 nm thick bottom Ag film (supplementary Fig. 10). It shows that the thickness of Ag layer, MMA layer and Ge layer are crucial for realizing lowest ψ_{min} and extreme phase singularity at the point of darkness. Based on our experimental analysis, the optimized thickness required to achieve lowest ψ_{min} and extreme phase singularity are: top Ag (20 nm), MMA (522 nm), Ge (10 nm) and bottom Ag (80 nm).

Supplementary Note 3: X-ray photoelectron spectroscopy analysis

We have utilized thiophilic feature of silver surface to bound thiolated-biotin on the surface. The synthetic scheme for the preparation of thiol-functionalized biotin molecule is shown below:

Scheme showing preparation of biotin disulphide (1):

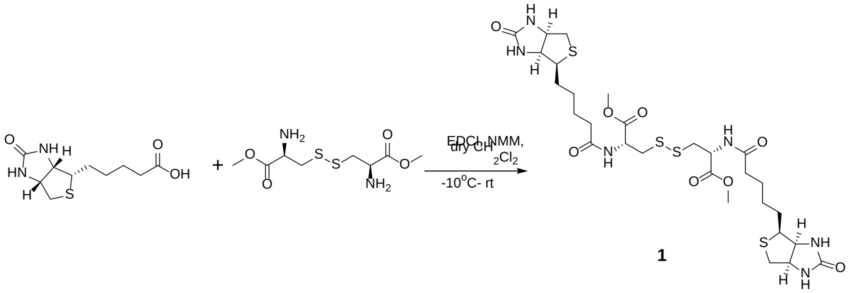

1

Scheme showing preparation of biotin thiol (2):

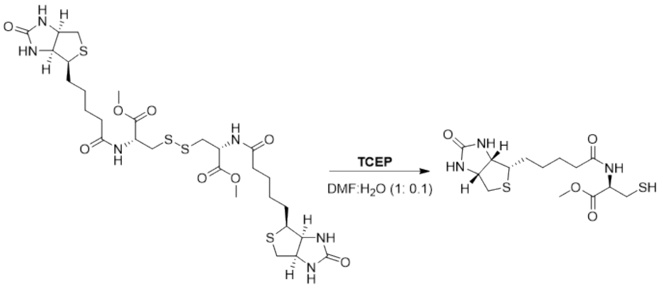

For ^{1}H NMR (Nuclear Magnetic Resonance spectroscopy) and mass spectrometry data of Product 2 are:

^{1}H NMR (CD_3OD, 400 MHz): δ = 1.45–1.52 (m, 2 H), 1.61–1.79 (m, 2 H), 2.13–2.24 (m, 1 H), 2.33 (t, 2 H, J = 4.0 Hz), 2.61–2.67 (m, 1 H), 2.72 (s, 3 H), 4.39–4.44 (m, 1 H, CH), 2.91 (d, 1 H, J = 8.0 Hz), 2.93–2.98 (m, 2 H), 3.15-3.25 (m, 2 H), 4.32-4.37 (m, 1 H), 4.50-4.53 (m, 1 H). MS (ES): m/z = 362.23 $[M + 1]^+$.

In the next step, we have conducted X-ray photoelectron spectroscopy (XPS) to confirm the effective functionalization of biotin-thiol utilizing Ag-S linkage. Supplementary Fig. 12a shows XPS survey spectrum of bare sample (top Ag layer). High-resolution XPS analysis and the peak

at 368.2 eV region indicates metallic Ag on the surface (Supplementary Fig. 12b). Measurements were carried out at multiple regions to confirm the observation and presence of no organic traces were recorded from bare sample.

The sensor surface (top Ag layer) was then modified using biotin-thiol (as described in the Methods section) and ensure complete incubation on the surface. The excess biotin-thiol were washed thoroughly using PBS buffer and samples were analyzed immediately after 12 h freeze-drying process. Supplementary Fig. 13a shows XPS survey spectrum of thiolated biotin containing Ag. Supplementary Fig. 13b shows a shift in the binding energy to 368 eV, which indicates the effective surface change due to the attachment of electron donating ligand (here thiol functional group from biotin-thiol). Similarly, XPS analysis further revealed peaks in the region corresponding to C 1s (supplementary Fig. 13c), N 1s (supplementary Fig. 13d), O 1s (supplementary Fig. 13e) and S (supplementary Fig. 13f). The presence of elements C, N, O and S on the surface further confirms the functionalization of the surface using biotin-thiol.

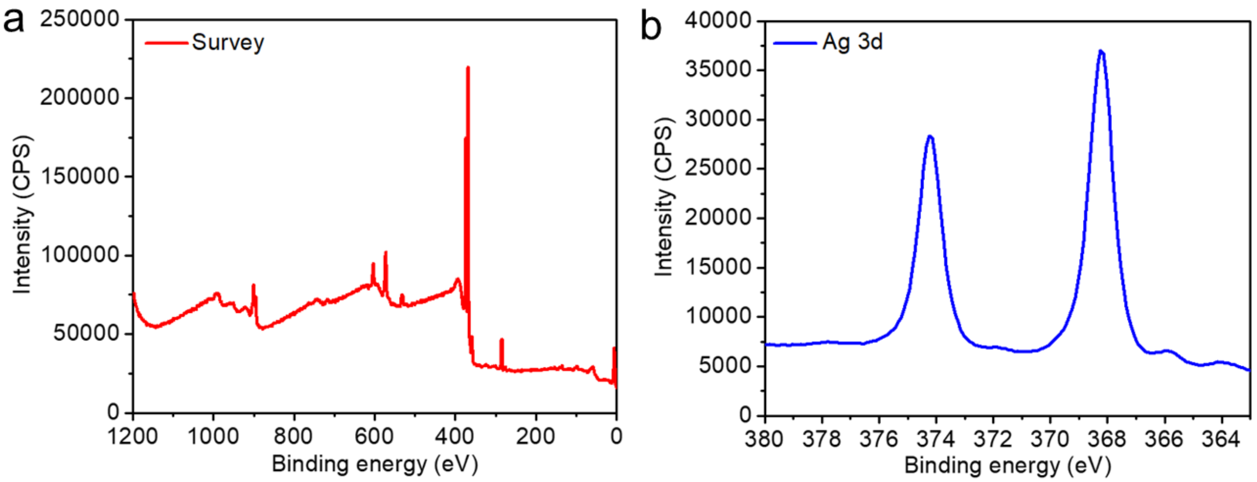

Supplementary Figure 12 | XPS spectrum of Ag surface without any functionalization. (a) survey, (b) peaks corresponding to metallic Ag (Ag 3d).

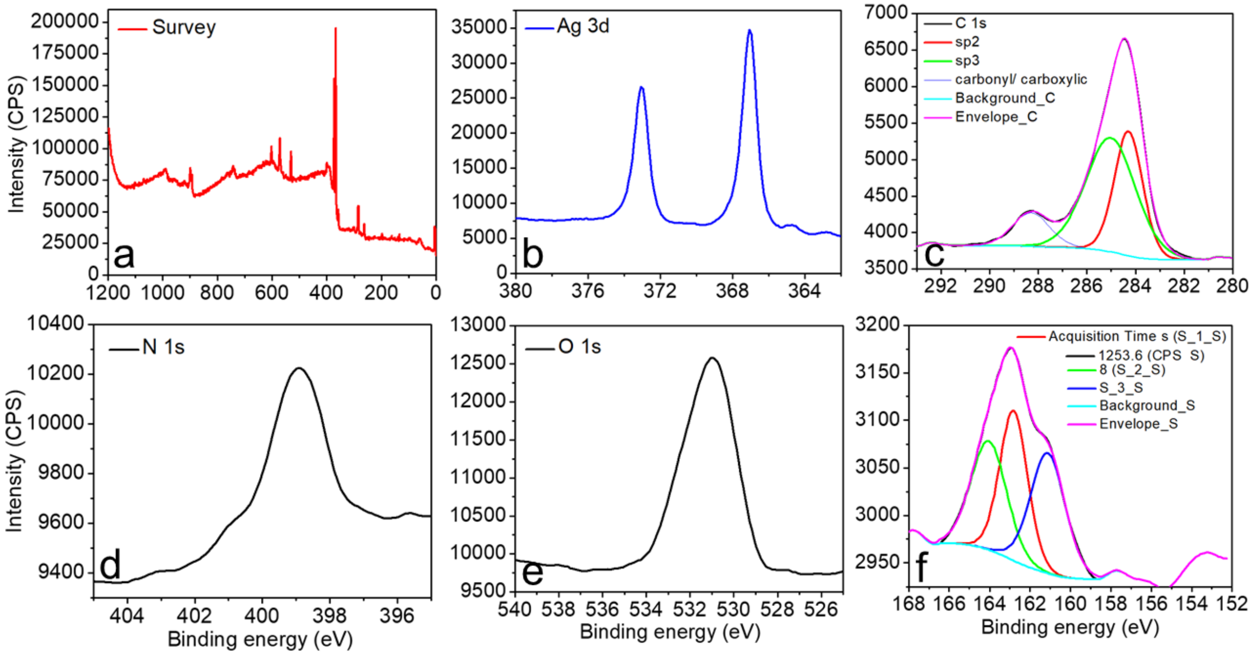

Supplementary Figure 13 | XPS spectrum of Ag surface after modification with biotin-thiol. (a) Survey, peaks corresponding to region of (b) metallic Ag (Ag 3d), (c) C 1s, (d) N 1s, (e) O 1s and (f) S.

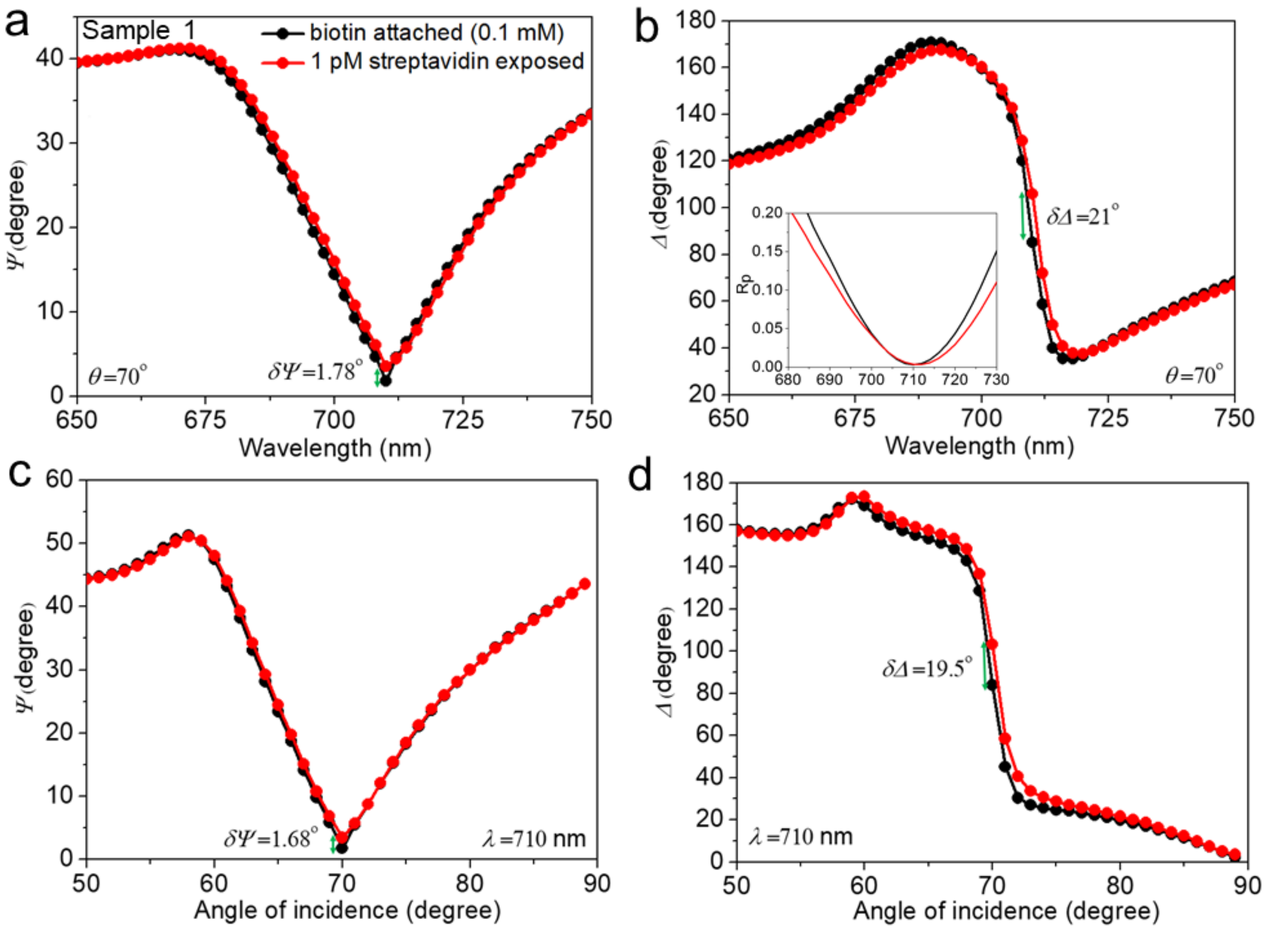

Supplementary Figure 14 | Biosensing data for sample 1. Ellipsometry parameters (a) ψ and (b) Δ versus wavelength before (black curve) and after (red curve) exposing 1pM streptavidin (at 70°). Ellipsometry parameters (c) ψ and (d) Δ versus incident angle before (black curve) and after (red curve) exposing 1pM streptavidin (at 710 nm).

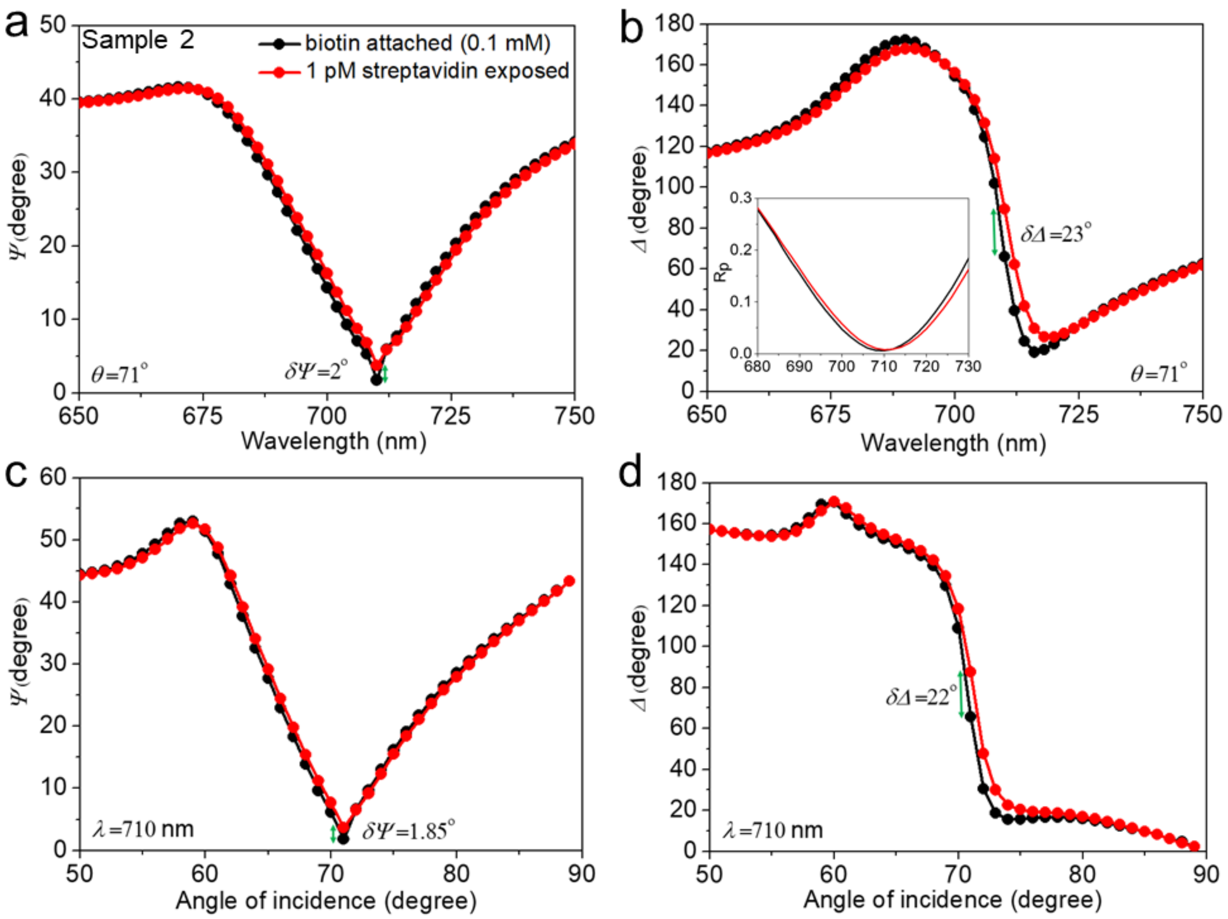

Supplementary Figure 15 | Biosensing data for sample 2. Ellipsometry parameters (a) ψ and (b) Δ versus wavelength before (black curve) and after (red curve) exposing 1pM streptavidin (at 71°). Ellipsometry parameters (c) ψ and (d) Δ versus incident angle before (black curve) and after (red curve) exposing 1pM streptavidin (at 710 nm).

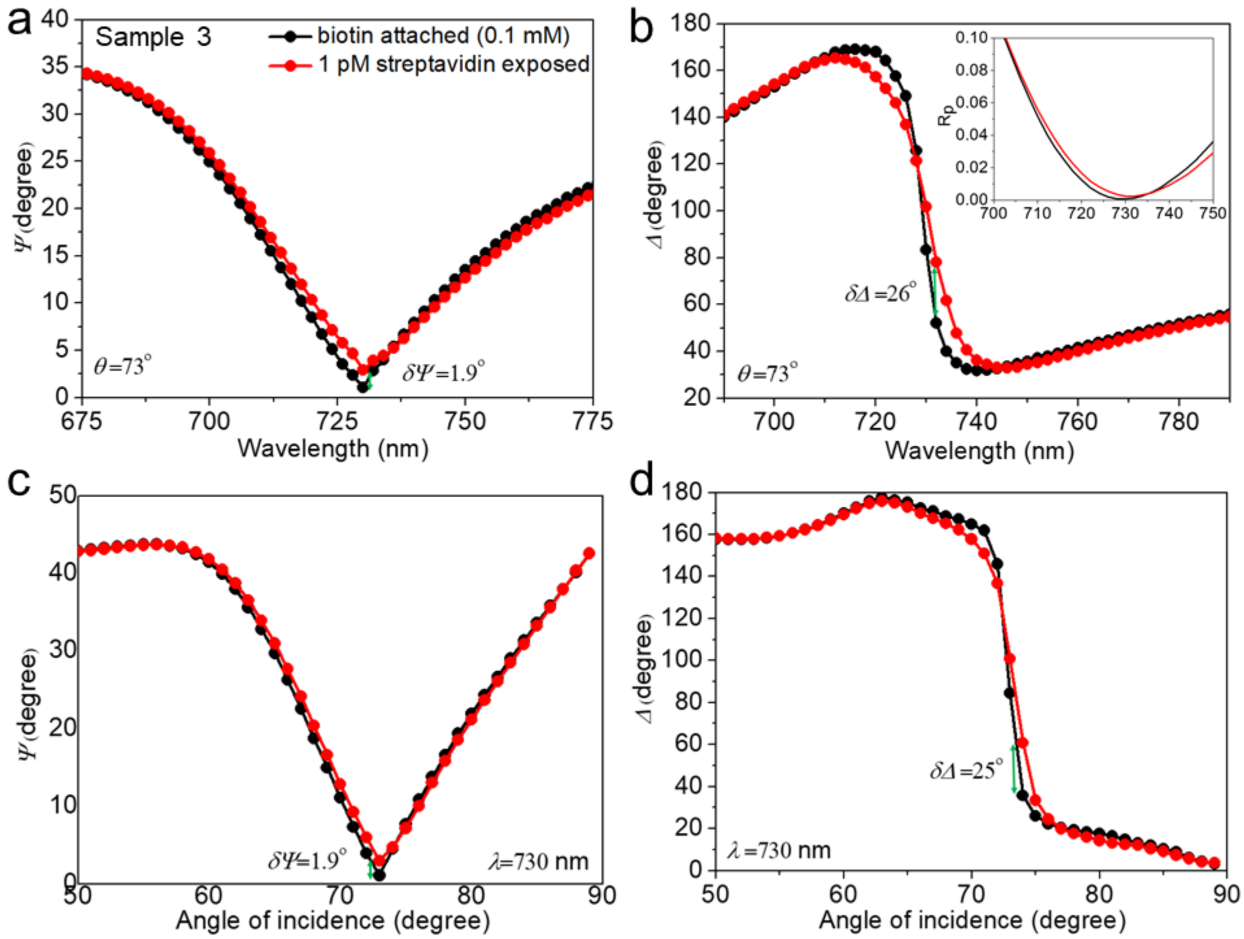

Supplementary Figure 16 | Biosensing data for sample 3. Ellipsometry parameters (a) ψ and (b) Δ versus wavelength before (black curve) and after (red curve) exposing 1pM streptavidin (at 73°). Ellipsometry parameters (c) ψ and (d) Δ versus incident angle before (black curve) and after (red curve) exposing 1pM streptavidin (at 730 nm).

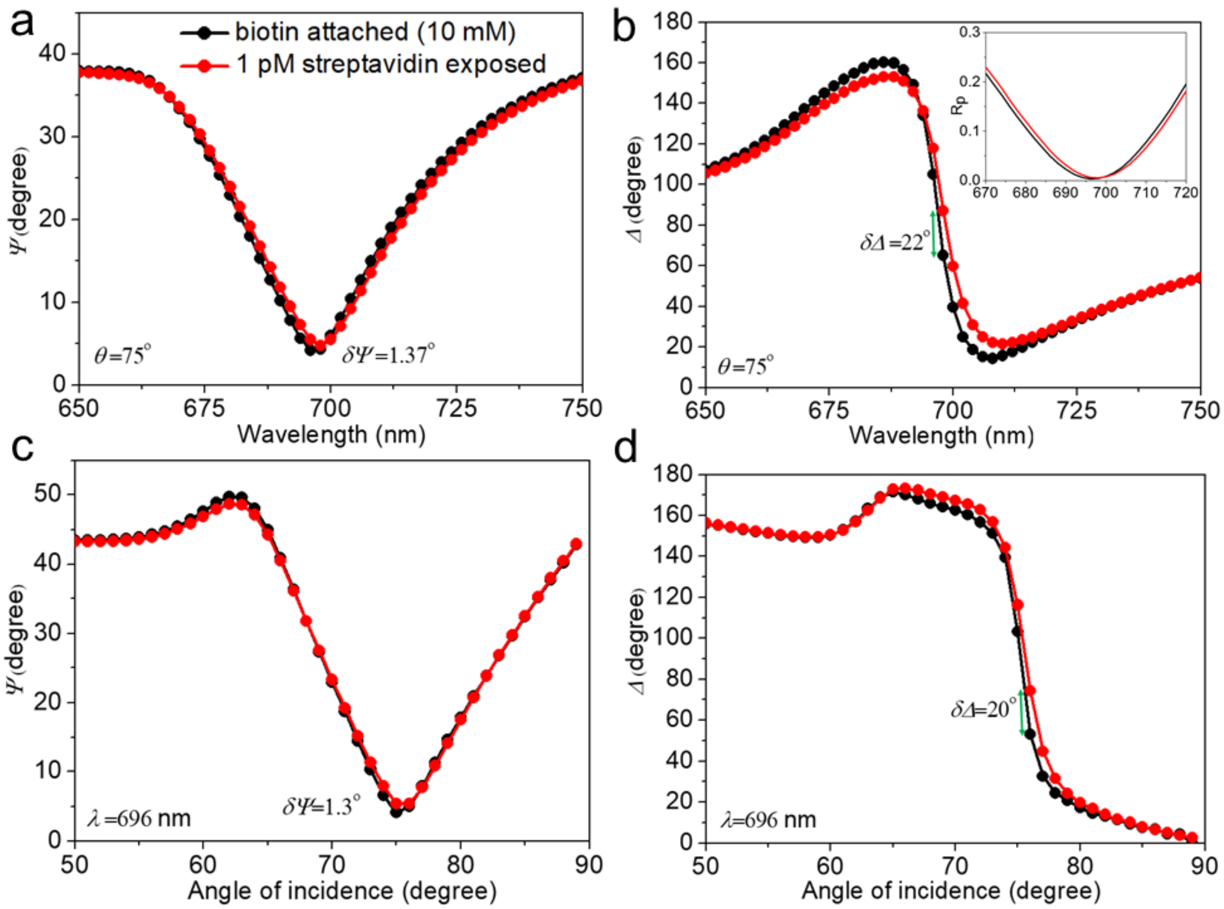

Supplementary Figure 17 | Biosensing data for 10 mM biotin attached sample. Ellipsometry parameters (a) ψ and (b) Δ versus wavelength before (black curve) and after (red curve) exposing 1pM streptavidin (at 75°). Ellipsometry parameters (c) ψ and (d) Δ versus incident angle before (black curve) and after (red curve) exposing 1pM streptavidin (at 696 nm).

Supplementary Note 4: Biosensing analysis

We have repeated sensing experiments many times and results obtained using three different samples from the same batch were presented here. Note that we cannot use the same sample for repeated measurements because it is not reusable once the biomolecules were captured. In order to keep lowest ψ_{min}, we have functionalized sample surface with 0.1 mM biotin-thiol (it can add a very thin layer of thickness approximately 1 nm on the sample surface). The experimental procedure is as follows: we first recorded the ellipsometry parameters (in both wavelength and angular scan) and *p*-polarized reflection spectra of biotin-thiol functionalized samples. Then, we have exposed 1 pM streptavidin in PBS for 2h and followed by rinsing with PBS to remove non-specifically bound streptavidin molecules using a detachable PDMS channel with dimension 7 x 7 x 2 mm^3. This was performed without removing sample from the ellipsometer stage in order to keep the same incident beam position on sample so that we could extract the correct shift. Note that we have removed the PDMS channel while doing the measurements because no phase singularity behavior was visible when channel is present.

The measured spectra (black curves) of 0.1 mM biotin-thiol covered samples are shown in supplementary Fig. 14, Fig. 15 and Fig. 16. As can be seen, lowest ψ_{min} (a point of non-complete darkness) was obtained at higher incident angle (above 70°) and the wavelength corresponding to ψ_{min} is drastically blue shifted to below 730 nm. It is well known that resonance wavelengths blue shift with increase in angle of incidence (supplementary Fig. 3). This is also due to the presence of a relatively rough thin layer of biotin-thiol. Also, in the case of top Au layer sample (supplementary Fig. 20), ψ_{min} is obtained at 718 nm due to lossy rough surface of Au layer.

As shown in supplementary Fig. 14 to Fig. 16, the spectral wavelength and angle at which lowest ψ_{min} obtained was varied from sample 1 to sample 3. This is due to the slight variation in surface roughness after functionalizing the samples with biotin-thiol. The lowest ψ_{min} obtained for sample1, sample2 and sample 3 were 1.78° at 710 nm and 70° (supplementary Fig.14), 1.74° at 710 nm and 71° (supplementary Fig. 15) and 1.03° at 730 nm and 73° (supplementary Fig. 16), respectively. It shows that ψ_{min} increased and phase singularity decreased after functionalizing the samples with biotin-thiol. Since it is not directly possible to use the point of darkness for sensing purpose due to the absence of light and abrupt phase jump, the obtained close to zero reflection condition (ψ_{min}=1° to 3°) is sufficient for biosensing.

The ellipsometry parameters recorded after exposing 1 pM streptavidin are shown as the red curves in supplementary Fig. 14 to Fig. 16. Almost same spectral sensitivity (wavelength red shift) in *p*-polarized reflection spectra and ψ spectra were obtained (supplementary Fig. 14b, Fig. 15b and Fig. 16b), which represents the refractive index change due to binding of streptavidin molecules on the sensor surface. For all three samples, obtained ψ_{min} shift is within 1.7° to 2° in both wavelength and angular scan. However, phase shift varied from 21° to 26° for sample 1 to sample 3. It is evident that significant phase shift is obtained at ψ_{min} point in comparison to wavelength and ψ_{min} shift. If one can carefully look for the phase shift obtained for the sample 1 to sample 3, it is clear that phase shift increased with decrease in ψ_{min} value. That means, sample 3 provides maximum response because lowest ψ_{min} (1.03°) was recorded for sample 3. We have recorded a maximum Δ change of ~26° at 73° angle of incidence. Therefore, we can also say that maximum phase shift is possible when ψ_{min} is obtained at higher angle of incidence. Since the lowest ψ_{min} is obtained at 73° for sample 3, it provides maximum phase shift whereas the corresponding angle for sample 2 and sample 1 were 71° and 70°, respectively.

Supplementary Note 5: Streptavidin binding study

In this section, we present a simple approach to understand the specific binding of streptavidin molecules on biotin functionalized surface. Since the phase sensitivity of the proposed sensor depends on $\psi_{\min}$ value, we slightly change the effective thickness of adsorbed streptavidin layer. In our approach, we keep the analyte (streptavidin) concentration as 1 pM in PBS buffer and change the functionalization biotin concentration in PBS buffer from 0.1 mM to 10 mM. It is well known in streptavidin-biotin model that the effective thickness (height) of adsorbed streptavidin layer depends on various parameters such as base binding groups, cleanliness of the surface and concentration of the functionalized molecules[2-4]. Therefore, we can expect a slight increase of effective thickness as the biotin concentration increases. This slight increase of effective thickness strongly effects $\psi_{\min}$ value and phase sensitivity.

The biosensing results obtained using 0.1 mM, 1 mM and 10 mM biotin concentration are shown in supplementary Fig. 16, Fig. 4 (in the manuscript) and supplementary Fig. 17, respectively. Note that we have repeated the experiments for each concentration using three samples and results of the samples in which maximum phase sensitivity obtained are shown here. It is evident from the figures that $\psi_{\min}$ is increased as the concentration of biotin is increased. The recorded $\psi_{\min}$ for 0.1 mM, 1mM and 10 mM biotin concentration are 1.03°, 2.5° and 4.1°, respectively. That means, lowest $\psi_{\min}$ is obtained for 0.1 mM biotin covered sample because 0.1 mM biotin only add an extra layer thickness <1 nm on the bare sample. On the other hand, the surface roughness is less compared to other samples. Therefore, phase sensitivity could be higher for 0.1 mM biotin covered sample because of lowest $\psi_{\min}$ value. A slight increase of $\psi_{\min}$ value ($\delta\psi$=1.9° for 0.1 mM and $\delta\psi$=5.9° for 1 mM) after exposing 1 pM streptavidin shows the

specific binding of streptavidin on the biotin sites. That means the effective thickness slightly increased after exposure.

The recorded maximum phase sensitivity after exposing 1 pM streptavidin on 0.1 mM and 1 mM biotin covered sample was 26º and 33º, respectively (see supplementary Fig. 16 & Fig. 4 (in the manuscript)). Notably, 1 mM biotin sample provided slightly higher phase sensitivity due to the availability of large number of biotin binding sites on the sample. Since it is well known in streptavidin-biotin system that four biotin molecules bind to one streptavidin molecule, more biotin molecules are available for streptavidin to bind in 1 mM biotin sample as compared to 0.1 mM biotin sample. The observed ψ change ($\delta\psi$=5.9°) also confirms that specific binding is strong in the case of 1 mM biotin. However, in the case of 10 mM biotin covered sample weak phase sensitivity was obtained because ψ_{min} value was largely enhanced to 4.1° (see supplementary Fig. 17). In addition, as the concentration increases, the possibility of multiple adsorbed molecules leads to interference effects, with each additional molecule having a decreasing impact on the phase shift.

It is also clear from *p*-polarized reflection spectra that slightly higher spectral wavelength shift (2 nm) is obtained for 1 mM biotin attached sample as compared to 0.1 mM biotin attached sample (1.6 nm). These results further confirm that streptavidin specific binding increases as the biotin concentration is increased from 0.1 mM to 1 mM. Since we have obtained different ψ change and Δ change with biotin concentration, and consistent red wavelength shift and positive angular shift after exposing streptavidin, observed sensitivity of the sensor is not due to bulk refractive index change and temperature drifts. As mentioned above, we have rinsed with PBS to remove non-specifically bound streptavidin molecules using a detachable PDMS channel. This procedure helps us to avoid the unspecific adsorption to a major extent. Therefore, the phase

sensitivity achieved for the proposed sensor is mainly due to specific adsorption (refractive index changes due to specific binding of streptavidin on the sensor surface). Since the demonstration of real-time binding of streptavidin over time is not so straightforward in our case, the proof-of-concept approach demonstrated here is appropriate for providing a more convincing proof of the specific binding of streptavidin.

Supplementary Note 6: Estimation of device sensitivity

To obtain the device sensitivity, we estimate the sensitivity of the phase shift to the number of molecules adsorbed on the sensor surface. In general, for each concentration c of the biomolecule in a device, there will be a corresponding saturating shift (wavelength, angle, intensity and phase), which is due to the presence of an average equilibrium population $N(c)$ of adsorbed particles on the sensor surface, a number which we cannot directly measure. In our case, device sensitivity can be defined as $\delta\Delta(c)/ N(c)$, or mean phase shift per adsorbed particle.

Since $N(c)$ is not straightforward to determine, we can estimate a reliable upper bound on this number, which is $N_{\max}(c)$, the maximum number of molecules on average that can be adsorbed on the sensor surface. Since $N(c) \leq N_{\max}(c)$, $\delta\Delta(c)/N_{\max}(c)$ will be a lower bound on the true sensitivity $\delta\Delta(c)/ N(c)$. In order to estimate $N_{\max}(c)$, we consider several factors that contribute to $N(c)$ such as illuminated beam area on the sensor, adsorption on boundaries of channel volume and irreversibility of adsorption.

Since the illuminated beam diameter is around 1mm^2, the effective sensor area is 1 mm^2. Note that only a small fraction of total population of streptavidin molecules will end up adsorbed on the illuminated sensor area. Adsorption can occur along the sensor surface and on the PDMS top and side surfaces of the channel. For $N_{\max}$, we will assume that only the sensor surface is adsorbing, since any competition from the PDMS surfaces will always lead to fewer molecules

on the sensor. We will also assume that the adsorbed molecules are equally distributed across the entire sensor surface, which has a dimension of 7 mm x 7 mm, an area of 49 mm^2. Hence, given a certain maximum possible adsorbed population on the surface, only a fraction 1 mm^2 /49 mm^2 = 0.02 (2%) will be in the sensing region and relevant to the phase shift. In principle, the equilibrium adsorbed population *N* reflects the net flux of molecules binding to the sensor areas minus the flux of molecules unbinding. To obtain, N_{max} we will assume that binding is irreversible, since any unbinding events will always be lower than adsorbed number.

Now we can calculate N_{max}. Since the PDMS channel has a height of 2 mm, initially there are c (7 mm x 7 mm x 2 mm/1L) M^{-1} = 5.9 c x 10^{19} M^{-1} biomolecules in solution inside the device. In the long-time limit, if all of these were to be adsorbed irreversibly on the sensor surface, on average 2% of the total would be in the sensor areas. Thus:

$$N_{max}(c) = 11.8\, c \times 10^{17}\ M^{-1} \qquad (1)$$

For 1 pM streptavidin solution, $N_{max} = 11.8 \times 10^5\ M^{-1}$

Since the obtained phase shift for 1 pM streptavidin solution using 1 mM biotin attached sample was 33° (see Fig. 4 in the manuscript), the estimated device sensitivity is, $\delta\Delta / N_{max} = 33°/ 11.8 \times 10^5 = 2.8 \times 10^{-5}$ degrees. The corresponding device sensitivity based on spectral wavelength shift ($\delta\lambda / N_{max}$) is 1.7 x 10^{-6} nm, where $\delta\lambda$=2 nm. It shows that phase sensitivity of the device is one order of magnitude higher than the spectral sensitivity, even though the direct comparison is not valid because of the unit difference.

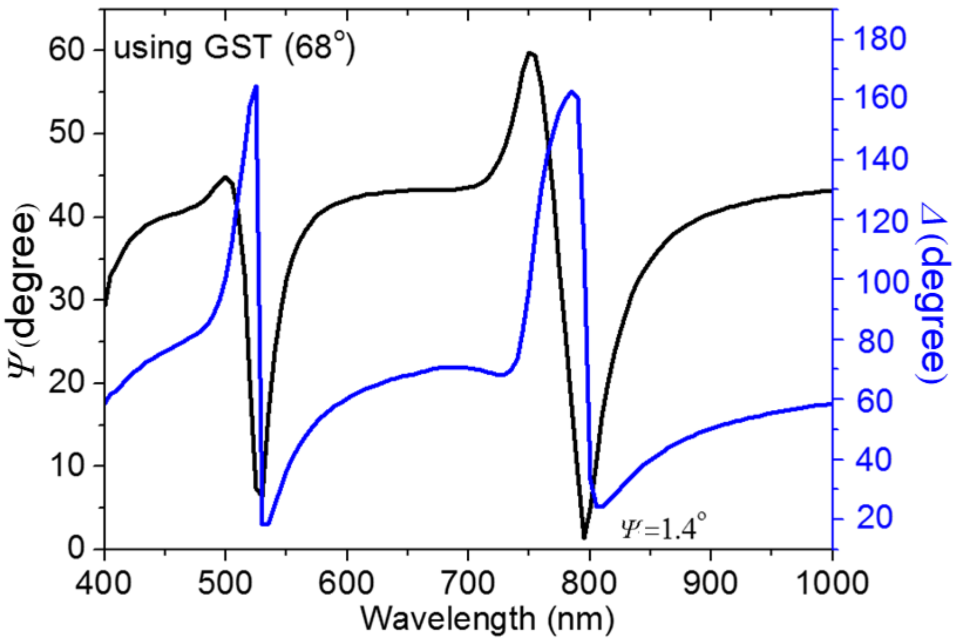

Supplementary Figure 18 | Ellipsometry parameters (*ψ* and *Δ*) of the proposed four-layered structure. Here Ge layer was replaced with 10 nm thick GST layer.

Supplementary Note 7: Realization of narrow cavity modes

Since the device is based on the principle of asymmetric Fabry-Perot cavity, the excitation of super narrow resonance mode is not straightforward. Note that both narrow resonance (high quality factor mode) and extreme singular phase are equally important for ultra-sensitive biosensing applications. Nevertheless, it is possible to achieve narrow resonance and singular phase by replacing Ge layer with another high index dielectric such as $Ge_2Sb_2Te_5$ (GST). Simulated ellipsometry parameters of the structure using 10 nm thick GST layer is shown in supplementary Fig. 18. Thickness of other layers used in the structure were: top Ag (20 nm), MMA (522 nm) and bottom Ag (50 nm). As can be seen, here both modes show narrow resonance and longer wavelength mode shows singular phase behavior. In contrast to Ge layer sample, extreme singularity is not achieved for this system because the obtained ψ_{min} was greater than 1. However, it could be possible to achieve narrow resonance and extreme phase singularity

simultaneously, by optimizing the thickness of MMA layer, GST layer and Ag layer. In addition, a tunable point of darkness condition and singular phase is possible for this geometry, by switching the phase of the GST from amorphous to crystalline.

Supplementary Note 8: Issues related to oxidation of top Ag surface

To check the oxidation issues of top Ag surface, we have recorded the ellipsometry spectra of bare sample twice, two days and 30 days after fabrication. The ellipsometry spectra of bare sample recorded for both times are shown in supplementary Fig. 19. As can be seen, no considerable change in resonance wavelength and incident angles were obtained after 30 days. However, ψ_{min} is slightly increased to 0.401° after 30 days (first time it was 0.26 ° in wavelength scan), which could be due to oxidation of thin Ag surface. Note that this slight increase of ψ_{min} not at all affect the biosensing performance because close to zero reflection condition (ψ_{min}=1° to 3°) is sufficient for biosensing. As mentioned above, ψ_{min} slightly increases after functionalization of the surface with biotin, therefore the oxidation of top Ag layer is not very critical here. We have also recorded XPS spectra both times. However, we did not identify any peaks related to oxygen in XPS analysis before and after biotin-thiol functionalization. It could be due to the mergence of oxygen peaks with some other elemental peaks. It further confirms that oxidation does not affect the functionalization of biotin on the sensor surface.

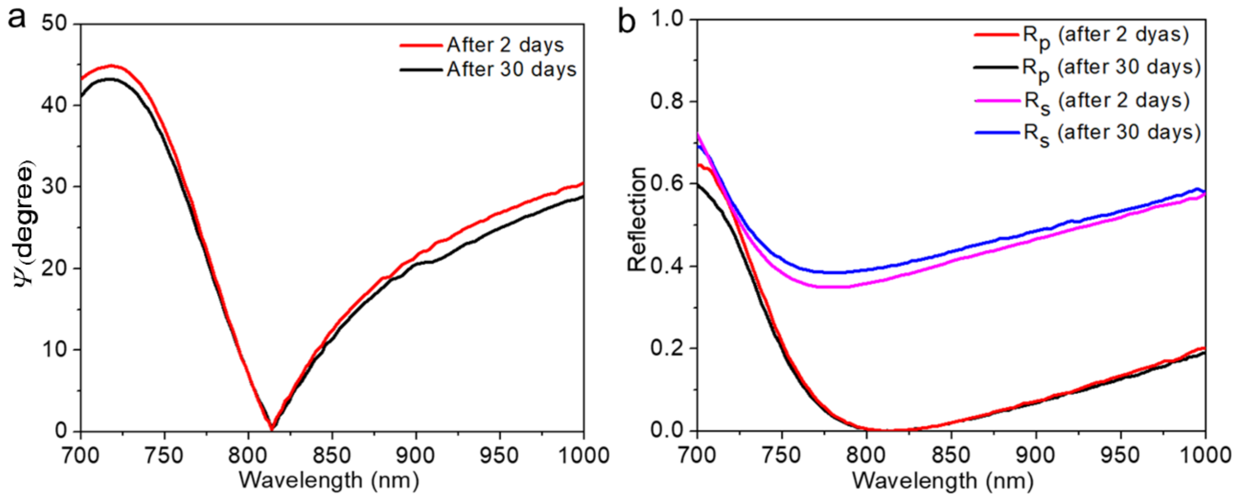

Supplementary Figure 19 | Twice recorded spectra at 61° angle of incidence stretched over a period of 2 and 30 days. (a) ψ spectra and (b) *p*- and *s*-polarized reflection spectra.

In order to study the top metal film effect on the ellipsometry parameters, we have replaced top Ag film with thermally evaporated gold film and recorded the data for different thickness of gold layer. The lowest ψ_{min} was obtained for an optimized top Au layer thickness of 19 nm. The ellipsometry parameters obtained at 65° angle of incidence are shown in supplementary Fig. 20. As shown in supplementary Fig. 20b, a lowest ψ_{min} of 4.1° and small phase change was obtained for the optimized geometry. The experimental and simulated reflection spectra for *p*- and *s*-polarizations are shown in supplementary Fig. 20c and 20d, respectively. Both experimental spectra were in good agreement with simulated spectra. It shows that selection of top metal layer is crucial to achieve ψ_{min}<1 and maximum phase change. Since silver thin film is low-loss compared gold thin film, silver is the best metal to realize point of darkness and extreme phase singularity. It shows that thermally evaporated gold film is not a good alternate for top Ag layer. Therefore, the potential alternative metal could be platinum.

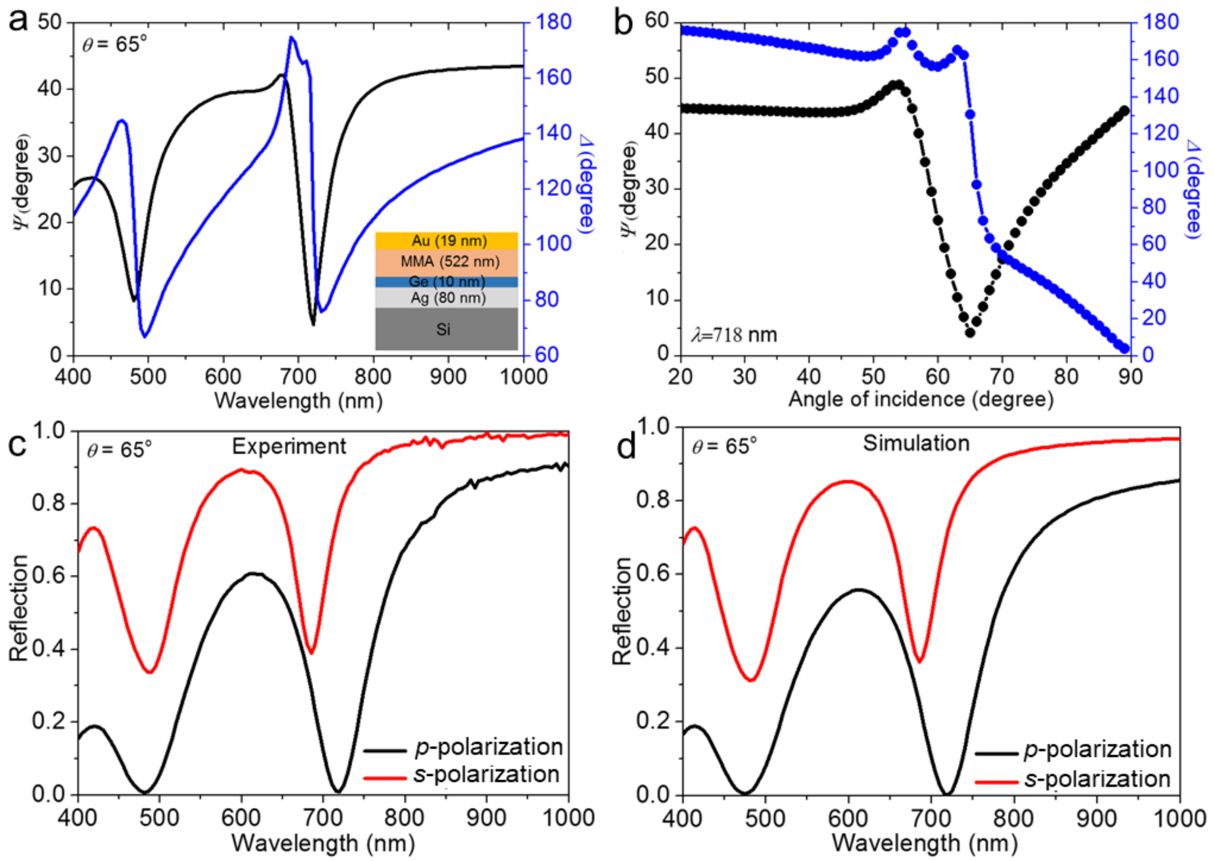

Supplementary Figure 20 | Experimentally determined ellipsometry parameters (*ψ* and *Δ*) and reflection spectra for samples with top metal layer as gold. (a) Wavelength scan spectra recorded at 65° (b) Angular scan spectra recorded at 718 nm, (c) experimental *p*-and *s*-polarized reflection spectra at 65° and (d) simulated *p*-and *s*-polarized reflection spectra at 65°.

Supplementary References

1. Mahlein, H. F. Generalized Brewster angle conditions for quarter-wave multilayers at non-normal incidence. *J. Opt. Soc. Am.* **64**, 647-653 (1974).
2. Neish, C. S., Martin, I. L., Henderson, R. M. & Edwardso, J. M. Direct visualization of ligand-protein interactions using atomic force microscopy. *British Journal of Pharmacology* **135**, 1943-1950 (2002).
3. Kohler, M., *et al*. pH-dependent deformations of the energy landscape of avidin like proteins investigating by single molecule force spectroscopy. *Molecules* **19**, 12531-12546 (2014).
4. Williams, E. H. *et al*. Immobilization of streptavidin on 4H-SiC for biosensor development *Applied Surface Science* **258**, 6056– 6063 (2012).
